# Priorities for support service needs among individuals with spinal cord injury

**DOI:** 10.1038/s41393-025-01079-9

**Published:** 2025-04-30

**Authors:** Hye Jin Nam, Haesun Lee, Ju Young Yoon

**Affiliations:** 1 College of Nursing, Seoul National University, Seoul, South Korea; 2 Research Institute of Nursing Science, Seoul National University, Seoul, South Korea

**Keywords:** Rehabilitation, Health policy

## Abstract

**Study design:**

Cross-sectional survey.

**Objectives:**

This study aims to identify and prioritize the gaps between the required and current levels of support services for individuals with SCI in South Korea.

**Setting:**

Community.

**Methods:**

The cross-sectional analysis involved 532 participants who are members of the Korea Spinal Cord Injury Association. These participants were recruited through the 2021 Survey on Needs of People with SCI, conducted via face-to-face interviews by trained investigators. The survey assesses the demand and current levels of 11 key support services. The Borich needs assessment and the locus for focus model were employed to identify the priorities of these services.

**Results:**

Results indicated significant disparities between the required and current levels across all services. Among the 532 participants, 70.1% were male and the average age was 49.6 years. The highest priority services were identified as “support for caregiver costs,” “livelihood security,” “health management,” and “home modification”. Secondary priorities included “training for daily living activities” and “vocational rehabilitation.”

**Conclusions:**

The study emphasizes the importance of aligning service provision with the specific needs of the SCI population to enhance their quality of life and promote successful reintegration into the community. The findings underscore the urgent need for comprehensive support systems to address the economic and functional challenges of individuals with SCI. Policymakers and service providers are encouraged to focus on these identified priorities to better meet the needs of individuals with SCI, to ensure their sustained independence and improved well-being.

## Introduction

Support services are crucial for individuals to achieve positive outcomes following a severe illness or injury, particularly for individuals living with permanent disabilities and complex needs, such as SCI [1–3]. Support services help ensure that individuals can remain independent despite chronic health issues, and can continue their education, work, and other meaningful life activities [4]. According to the World Health Organization, 2.4 billion people, or one in three globally, will experience a health condition in their lifetime that would benefit from support services [5, 6]. However, the demand for support services remains largely unmet [4]. For individuals with permanent disabilities, unmet needs for services can compromise health status and exacerbate the disability [7].

SCI is highly variable and multifaceted, and is often accompanied by secondary health complications [8]. Consequently, individuals with SCI are likely to require a range of mainstream and specialized services to effectively address their needs [9]. Support services for people with SCI encompass a wide range of therapies and support mechanisms designed to meet their diverse needs. For example, SCI rehabilitation programs are tailored to each individual’s requirements, aiming to restore body functions, develop strategies to compensate for impaired functions, utilize appropriate assistive devices, and remove environmental barriers [10]. Specifically, initial in-patient rehabilitation focuses on restoring physical functions and developing compensatory strategies through intensive therapies, including physical and occupational therapy, as well as psychological support [11, 12]. Ongoing community-based care includes outpatient rehabilitation, home modification services, and vocational training to support long-term independence and reintegration into society [11]. Grants and financial support are also available given the significant financial burden associated with SCI including loss of income, medical bills, rehabilitation costs, and daily care expenses. Specifically, South Korea offers a support system for individuals with SCI through its national health insurance, medical aid programs, and disability allowances. Eligibility for these programs depends on income thresholds and official disability registration status [13]. Income thresholds are based on household income levels compared to national poverty standards. Household classified as low-income or extremely low-income qualify for significant subsides or full coverage for medical services. Also disability registration requires an application, a medical diagnosis, and a disability evaluation by certified institutions, following guidelines set by the Ministry of Health and Welfare [14]. This registration categorizes individuals into grades, with higher grades (indicating more severe disabilities) granting access to a broader range of benefits, such as caregiver support, home modification assistance, and transportation subsidies. Rehabilitation services, including assistive devices and caregiver costs, are partially subsidized under these schemes but often require out-of-pocket contributions. These support services aim to meet the specific needs and goals of individuals with SCI to help maximize independence, improve quality of life, and facilitate reintegration into the community [10]. Thus, identifying and emphasizing the most demanded and most unmet services for individuals with SCI, representing services with the highest perceived gap between required and current levels, can lead to increased satisfaction and more effective outcomes for a higher quality of life [15].

To date, researchers have attempted to identify the service needs of individuals with SCI. Trezzini et al. [16] found that general healthcare and accessible housing were high-prevalence needs with a high level of fulfillment. Other studies have identified unmet service needs and the most critical service requirements for people living with SCI [7, 17]. However, they have not systematically reported the service gaps and priorities. A study by Alavinia and colleagues [18] employed a systematic priority-calculating formula to prioritize domains of SCI rehabilitation care. However, this prioritization was derived solely from 17 experts’ recommendations and did not directly reflect the perspectives of individuals with SCI. To address these limitations, this study draws from the 2021 Survey on Needs of People with SCI conducted by the Korea Spinal Cord Injury Association (KSCIA). This survey collected detailed information on the required and current levels of 11 key support services. It is crucial to systematically assess the priorities by comparing the required services with the extent to which current services meet the needs, with a focus on the experiences and expressions of individuals living with SCI. This approach will help identify the most urgent service needs and ensure that efforts are concentrated on areas that are most important to this population.

Support services are crucial for their ongoing journey including transitioning to a new lifestyle and learning how to participate in the community. The overarching goal of providing the necessary services is maximizing an individual’s physical, psychological, and social capabilities to attain meaningful life objectives. In line with this, individuals living with SCI have expressed a desire for increased involvement in their ongoing healthcare and support processes. Therefore, this study aims to identify the gaps between the required and current levels of support services and to identify the priority services for individuals with SCI. It is essential for policymakers and service providers to understand the perceived gaps in service delivery and identify the most critical service needs so they can offer appropriate services that support community living for individuals with SCI. By addressing these gaps, they can better meet the needs of this population to enhance their quality of life and promote successful reintegration into the community.

## Methods

### Study sample

This cross-sectional study analyzed secondary data from the 2021 Survey on Needs of People with SCI, collected by the KSCIA [19]. KSCIA serves as one of the primary organizations operating through a central headquarters in Seoul and regional branches across 16 administrative areas. KSCIA membership is open to all individuals with SCI, their families, and those involved in SCI-related support and advocacy. While not all individuals with SCI are members of KSCIA, the association’s structure are outreach efforts aim to address the diverse needs of the SCI population across Korea, promoting inclusivity and regional equity in its various programs. The association conducts a survey every three years to gather descriptive statistics on individuals with SCI. In-person surveys were conducted by trained investigators between May and June in 2021.

### Sample/Participants

The participants in this study are members of the KSCIA. According to KSCIA, participants were stratified to maintain an equal distribution across key demographic and injury-related characteristics. These included activity level, distinguishing between individuals who go out less than 4 times per month (excluding hospital visits) and those who go out more frequently; gender distribution, maintaining a male-to-female ratio of approximately (7:3); time since injury, categorized into three groups: less than 5 years, 6–10 years, and over 11 years; and injury site, ensuring a balanced representation of thoracic and cervical injuries [19].

### Variables

#### General characteristics

Demographic characteristics were recorded, including age, sex (male/female), education, monthly household income, marital status, time since injury, site of injury, and activities of daily living (ADL). Monthly household income was calculated based on equivalized income using the square root scale (i.e., total household income divided by the square root of the number of household members) [20]. The ADL assessment used in the 2021 Survey on Needs of People with SCI was developed by the Korea Spinal Cord Association to specifically address the functional status of people with SCI [19]. The questions included 11 items to assess the degree of difficulty performing ADL, such as eating and drinking, washing upper or lower body, dressing upper and lower body, grooming, bladder and bowel management, toileting, and short distance transfer (e.g., bed to wheelchair) or long distance transfer. ADL assessment is scored on a scale from 0 (no difficult at all) to 10 (a lot of difficulty), with a maximum possible score of 110. The Cronbach’s alpha of ADL was 0.95 in this study.

#### Support services

The extent of required and current levels of support services needs were measured using 11 services on an 11-point scale from 0–10. The support services included “medical rehabilitation,” “assistive devices,” “psychological counseling,” “family support,” “training for activities of daily living,” “home modification,” “livelihood security,” “vocational rehabilitation,” “health management,” “activity support,” and “support for caregiver costs.” The degree of demand is represented by the required level for each support service, while the degree of fulfillment of support service needs is represented by the present level. For example, a score of 10 on the required level indicates that the participant perceives the service as critically necessary, while a score of 10 on the present level indicates complete satisfaction with the current provision of the service. The reliability values were 0.91 for the required level of support services and 0.92 for the present level.

### Statistical methods

In this study, “needs” are defined as the discrepancy between participants’ expressed demand and current levels of support services. To assess priorities through needs analysis, statistical methods were adopted in accordance with the recommendations by Cho [21]. First, descriptive statistical analysis was employed using frequencies, percentages, means, and standard deviations to describe the sample characteristics and summarize the study variables. To examine the difference in the discrepancy between the required and current level of support services, we used general characteristics, t-test, ANOVA, and Pearson’s correlation coefficient. A paired t-test was also utilized to examine the discrepancy between the required and current levels of support services.

**Fig. 1:**
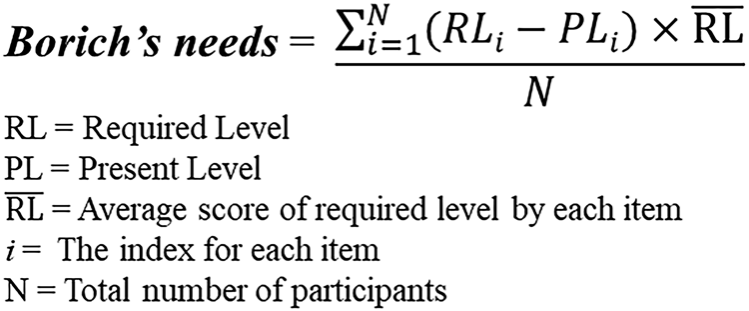
The formula for the priority decision of Borich needs.

Third, to address the inherent limitations of the simple difference comparison inherent in the paired t-test and to identify priority needs, we employed Borich needs assessment [22]. Figure 1 demonstrates the Borich needs assessment formula. In the Borich needs assessment, the required level pertains to the ideal state of the desired services, representing “what should be” based on participants’ preferences. The present level, on the other hand, reflects the current state of affairs as perceived by the participants in their current circumstances, indicating “what is.” It serves as an indicator of the level of satisfaction with their support service needs. As evident in the formula, the calculation places greater emphasis on the desired level compared to the current status. This approach offers the advantage of ranking needs according to the calculated scores.

Fourth, the locus for focus model (LFM) was utilized to visualize the priorities. The LFM visually represents the priorities using a coordinate plane, with the required level plotted on the horizontal axis (x-axis), and the discrepancy between the required level and present level plotted on the vertical axis (y-axis) [21, 23]. The median value on the horizontal axis represents the average of the required level, while the median value on the vertical axis represents the average discrepancy. In the first quadrant, termed the high-discrepancy, high-demand (HH) quadrant, both the discrepancy between the required level and present level, and the required level exceed the average. This quadrant represents the highest priority area for addressing support service needs among the four quadrants. The second quadrant, characterized by high discrepancy and low demand (HL), signifies the required levels that are lower than average but a discrepancy higher than average. This quadrant holds the second-highest priority as it is essential to address low demand while enhancing satisfaction. In the third quadrant, both discrepancy and demand are low. Finally, in the fourth quadrant, the discrepancy is minimal, but the required level is high. Although the LFM makes it easy to prioritize items in the first quadrant, it may be difficult to determine priorities even within the same quadrant [21].

In the final step, the service items in the first quadrant (HH) of the LFM with high priority in the Borich needs assessment, were assigned the highest priority. The items in the second quadrant (HL) were assigned the second-highest priorities.

### Ethical considerations

This study was a secondary analysis of cross-sectional survey data. The Korea Spinal Cord Injury Association has stringent protocols that ensure confidentiality and participant autonomy. Written informed consent was obtained from all subjects or their legal guardians. To ensure compliance with de-identified data handling procedures, our study was approved by the Institutional Review Board of Seoul National University (IRB No. E2405/004-012).

## Results

### General characteristics of participants

This study included 532 participants, among whom the majority was men (70%). The average age was 49.6 years (standard deviations, SD 11.7) and 31% were in their 40 s. Most participants graduated high school or above (43 and 43%, respectively). The mean household income was 16.5 million Korean Won (SD 183.8). More than half of the participants were single (58%). The average time since the spinal cord injury was 18.5 years (SD 11.5) and for over half of the participants, the main injury site was lower thoracic (27%) or lower cervical (26%). The ADL scores ranged from 0 to 110, with a mean of 63.7 (SD 30.5).

Table 1 shows the discrepancy between the required and present levels of support services based on general characteristics. Women showed a statistically significantly higher discrepancy than men (*p* = 0.009). The discrepancy decreased with increasing age (*p* = 0.010), while individuals with a university education reported a higher discrepancy compared to those with a middle school education or lower (*p* < 0.001). Furthermore, the discrepancy increased with higher monthly household income (*p* = 0.038). In terms of injury site, individuals with upper thoracic injuries exhibited a higher discrepancy compared to those with lumber or sacral injuries (*p* = 0.036). Lastly, individuals with greater independency in ADL demonstrated a higher discrepancy (*p* = 0.007). Marital status and time since injury showed no significant differences in the required and present levels of support services.

**Table 1:** General characteristics of participants and the discrepancy between the required and present levels based on general characteristics (*N* = 532).

Variables	Categories	n (%) or M ± SD	Required level	Present level	Discrepancy^a^		
			M ± SD	M ± SD	M ± SD	*t* or F^b^ or *r*	*p*
Sex	Male	373 (70)	91.5 ± 18.4	51.3 ± 24.5	40.2 ± 30.0	–2.61	0.009
	Female	159 (30)	94.9 ± 19.1	47.2 ± 22.7	47.7 ± 30.6		
Age (yr)	(range: 18–78)	49.60 ± 11.69				–0.11	0.010
	≤29	38 (7)	88.1 ± 23.8	41.5 ± 19.1	46.5 ± 34.5	1.65	0.146
	30–39	58 (11)	93.0 ± 17.1	49.4 ± 20.7	43.6 ± 24.9		
	40–49	167 (31)	95.6 ± 16.8	49.9 ± 25.3	45.7 ± 30.3		
	50–59	144 (27)	92.4 ± 17.6	50.5 ± 24.0	42.0 ± 31.0		
	60–69	109 (21)	90.9 ± 20.2	52.6 ± 24.4	38.2 ± 29.9		
	≥70	16 (3)	82.1 ± 80.8	53.6 ± 29.3	28.5 ± 32.2		
Education	≤Elementary school	32 (6)	88.1 ± 22.5	57.7 ± 22.8	30.3 ± 26.7	7.28	<0.001 ES, MS < Univ
	Middle school	47 (9)	84.1 ± 21.8	53.9 ± 23.2	30.3 ± 29.0		
	High school	227 (43)	93.7 ± 17.9	52.7 ± 23.6	41.0 ± 28.6		
	≥University	226 (43)	93.7 ± 17.5	45.6 ± 24.2	48.1 ± 31.5		
Household income (million KRW)	(range: 0–2828.43)	165.3 ± 183.8				0.09	0.038
Marital status^c^	Single	308 (58)	92.4 ± 19.1	49.7 ± 24.1	42.7 ± 30.8	0.164	0.870
	Married	224 (42)	92.8 ± 18.0	50.6 ± 24.1	42.2 ± 29.8		
Time since injury (yr)	(range: 1–68)	18.5 ± 11.5				0.08	0.081
Site of injury^d^	Upper cervical	81 (15)	88.8 ± 21.4	50.4 ± 22.2	38.3 ± 30.1	2.41	0.036 LS < UT
	Lower cervical	138 (26)	93.2 ± 16.6	47.9 ± 23.5	45.3 ± 28.9		
	Upper thoracic	93 (18)	97.1 ± 19.2	49.3 ± 25.1	47.9 ± 30.3		
	Lower thoracic	145 (27)	93.0 ± 19.2	50.3 ± 24.3	42.7 ± 31.2		
	Lumber/sacral	25 (5)	90.2 ± 17.7	61.2 ± 24.1	29.0 ± 29.6		
	Unclear	50 (9)	88.2 ± 22.5	50.7 ± 25.5	37.5 ± 30.4		
ADL	(range: 0–110)	65.7 ± 30.5				0.12	0.007

*ADL* activities of daily living, *M* mean, *SD* standard deviations, *yr* years, *KRW* Korean Won, *t* t-test, F = Analysis of Variance, r = Pearson’s correlation, *ES* Elementary school, *MS* Middle school, *Univ* University, *LS* Lumber/sacral, *UT* Upper thoracic.

^a^Discrepancy = Total demand scores – Total fulfilled scores in each persons.

^b^Scheffe post-hoc test.

^c^Including never married, divorced, separated, and widowed.

^d^if more than one site was injured, the main injured site was selected.

### Support services needs

Table 2 presents the findings of the paired t-test and Borich needs assessment. The paired t-test to evaluate the disparities between the required and present levels of each item concerning support services revealed significant differences for all 11 items (*p* < 0.001). Notably, the required level was consistently higher than the present level for all items.

**Table 2:** Differences in support service needs and the Borich needs assessment by items.

Item	Support services	Required level	Present level	Discrepancy^a^	*t*	Borich needs
		M ± SD	M ± SD	M ± SD		
A	Medical rehabilitation	8.80 ± 1.93	5.42 ± 2.57	3.37 ± 2.92	26.62^***^	29.68
B	Assistive devices	8.77 ± 2.04	5.50 ± 2.71	3.27 ± 3.14	23.97^***^	28.65
C	Psychological counseling	7.57 ± 2.71	4.05 ± 2.70	3.52 ± 3.54	22.98^***^	26.67
D	Family support	7.59 ± 2.67	4.09 ± 2.90	3.49 ± 3.85	20.91^***^	26.52
E	Training for daily living activities	8.24 ± 2.41	4.31 ± 2.98	3.93 ± 3.58	25.33^***^	32.38
F	Home modification	8.69 ± 2.22	4.57 ± 3.23	4.12 ± 3.84	24.73^***^	35.80
G	Livelihood security	8.68 ± 2.15	4.42 ± 2.95	4.26 ± 3.58	27.39^***^	36.94
H	Vocational rehabilitation	7.95 ± 2.70	3.71 ± 2.84	4.24 ± 3.62	26.99^***^	33.66
I	Health management	8.67 ± 2.06	4.41 ± 2.80	4.25 ± 3.42	28.67^***^	36.86
J	Activity support	9.03 ± 1.91	5.86 ± 3.15	3.17 ± 3.36	21.73^***^	28.62
K	Support for caregiver costs	8.56 ± 2.54	3.72 ± 0.320	4.84 ± 4.05	27.62^***^	41.47

*M* mean, *SD* standard deviations.

^***^p < 0.001.

^a^Discrepancy = Required level – Current level in each items.

After calculating the Borich needs assessment scores, “support for caregiver costs” emerged as the highest priority, with a score of 41.47. The next priorities were “livelihood security” (score 36.94), and “health management” (score 36.89). Conversely, the lowest priorities were “family support” (score 26.52), “psychological counseling” (score 26.67), and “activity support” (score 28.62).

**Fig. 2:**
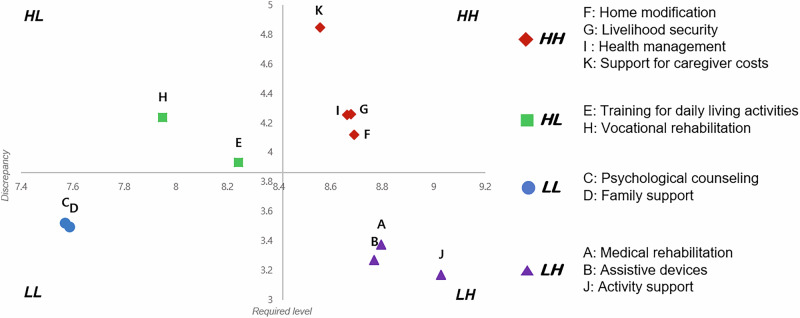
The locus for focus model for support services needs for people with spinal cord injury. HH high discrepancy with high demand, HL high discrepancy with low demand, LL low discrepancy with low demand, LH low discrepancy with high demand.

The results of visualizing the priorities using the LFM are depicted in Fig. 2. The analysis revealed an average required level of 3.86 on the horizontal axis for support services. Additionally, the mean discrepancy between the required and present levels was 8.41 on the vertical axis. In the first quadrant, representing high discrepancy with high demand, four items were identified: “home modification,” “livelihood security,” “health management,” and “support for caregiver costs.” Two items were in the second quadrant, signifying high discrepancy with low demand, and another two items were in the third quadrant, characterized by low discrepancy and low demand.

To determine the highest priority items, we employed a combination of the Borich needs assessment and LFM. Four items from the top priorities identified in the Borich needs assessment were selected, aligning with the four items in the first quadrant as determined by the LFM (Table 3). The top priority items identified through both the Borich needs assessment and the LFM were found to be consistent. As a result, “support for caregiver costs” emerged as the highest priority, followed by “livelihood security,” “health management,” and “home modification.” Furthermore, two items in the second quadrant, “vocational rehabilitation” and “training in activities of daily living,” were also matched as the second top priorities according to the Borich needs assessment.

**Table 3:** Priorities of support service needs based on the Borich needs assessment and the locus for focus model.

Items	Support services	Borich needs rank	The locus for focus model	Priority
K	Support for caregiver costs	1	HH	Highest
G	Livelihood security	2	HH	Highest
I	Health management	3	HH	Highest
F	Home modification	4	HH	Highest
H	Vocational rehabilitation	5	HL	Second-highest
E	Training for daily living activities	6	HL	Second-highest

*HH* high discrepancy with high importance, *HL* high discrepancy with low importance.

## Discussion

The present study prioritized support service needs of individuals with SCI in South Korea using Borich needs assessment and the LFM. Previous researchers have attempted to explore the demand and unmet needs for support services for individuals with SCI, responding to the growing calls for such investigations [16, 24, 25]. This study takes a novel approach to comprehensively assess the priorities to gain insights into the specific types of support services that individuals with SCI most urgently require. Among the 11 types of services, this study uncovered four services that individuals with SCI ranked as the highest priorities, and two services as the second highest priority services. Notably, we observed a significant disparity between the level of required support services and the actual fulfilled level of support services across all categories, indicating a widespread shortfall in meeting the needs of the SCI population. Taken together, these results suggest that individuals with SCI have diverse support service needs that may not be adequately addressed.

“Support for caregiver costs” was recognized as one of the highest priority support services for individuals with SCI. A national report on SCI in South Korea indicated that caregiver costs account for 21.9% of the extra costs of living with a SCI [20]. This underscores the significant need for caregiver support, leading to high costs associated with this condition [26]. It is estimated that 40% of all individuals with SCI require some level of assistance with personal care [27]. Approximately 61% of the care is paid assistance, with the remainder provided by family members or other informal caregivers [28]. Family caregivers often reduce their working hours or stop working entirely to accommodate the demands of caregiving demands [29]. Relying on the unpaid labor of family caregivers is a global issue for healthcare and social systems. To ensure their continued involvement, policymakers must explore avenues to recognize and potentially compensate informal caregivers for their invaluable contributions. Beyond financial support, enhancing community-based caregiving programs, expanding training for informal caregivers, and fostering partnerships with private entities could provide sustainable solutions without solely relying on subsidies.

Our study identified “livelihood security” as another high-priority service, underscoring the profound economic difficulties individuals with SCI face. It is widely recognized that people with disabilities, including SCI, are more susceptible to higher levels of deprivation and poverty, often associated with the extra costs of living with a disability [30]. Significant expenses are incurred over the lifespan of individuals with SCI, including initial hospitalization, acute rehabilitation, home and vehicle modifications, as well as recurring costs for durable medical equipment, medications, supplies, and personal assistance [31]. They also experience the loss of wages, fringe benefits, and productivity related to unemployment after SCI [32]. In essence, these expenses are a burden not only for the individuals with SCI, but also for the family members who are responsible for their care and on society as a whole [33]. Individuals with SCI are living longer compared to historical trends [34], so the condition imposes a significant financial burden over the course of their lives, beginning in the acute care setting. In the context of South Korea, while various financial benefits and support system exist, our findings imply gaps in their design and accessibility. For example, eligibility criteria based on income thresholds any inadvertently exclude individuals who still face substantial economic burdens due to the high cost associate with SCI. In South Korea, various financial benefits and support systems are available for individuals with disabilities, including national health insurance and medical benefits, discounts and exemptions for public charges, basic benefits to supplement low-income individuals, and a disability allowance [35]. However, as indicated by the high priority placed on “livelihood security” and “support for caregiver costs”, these support systems may not sufficiently alleviate the additional economic burden faced by individuals with SCI. While existing financial support system in South Korea provide assistance through various benefits and subsidies [35], our findings underscore gaps in accessibility and adequacy for individuals with SCI. Targeted interventions could focus on ensuring better alignment between eligibility criteria and the diverse economic realities faced by individuals with SCI. Additionally, further research into the effectiveness of these existing programs and their reach to diverse income groups could provide valuable insights into optimizing support.

Another high-priority support service was “health management” for individuals with SCI. The identified gaps in care include preventive measures such as cancer screening and immunizations, management of bladder and bowel functions, skin issues, sexual health, and pain management [36, 37]. Moreover, psychological disorders such as depression, anxiety, and substance abuse are prevalent but frequently under-recognized [38]. Many health issues individuals with SCI experience could improve with access to quality primary care [39]. However, while physical inaccessibility to offices and equipment often pose challenges for individuals with disabilities, the most common barrier for patients with SCI is effectively accessing first-line primary care providers with specialized knowledge and expertise [40]. Cox et al. [25] revealed that 81% of individuals with SCI perceive that local providers have limited knowledge about SCI and report that it is the primary barrier to meeting their healthcare needs. Another study indicated that individuals with SCI rely heavily on peer support networks for health advice, highlighting the need for accessible and comprehensive healthcare services with knowledgeable providers [41]. To address these challenges, in 2018, the Korean government initiated a pilot project to assign disability-specialized general practitioners to provide primary care for people with disabilities [42]. Despite these efforts, the utilization rate of this service remains low, accounting for less than 1% of the disabilities population [42], emphasizing the need for awareness campaigns and evaluations to identify barriers. There is an urgent need for the government to invest in training and education programs to enhance providers’ expertise in SCI care. It is also crucial to foster collaboration among government agencies, healthcare providers, disability organizations, and community stakeholders. This collaboration is essential for developing and implementing effective strategies to improve medical accessibility and continuity of care for individuals with disabilities.

The final high-priority support services need was “home modification.” This priority emphasizes the crucial role of housing accessibility for individuals with SCI to promote independence, support health, and enhance occupational performance [43]. Research has also shown that restoring mobility after an injury is a top life priority for individuals with newly acquired SCI [44]. In particular, mobility barriers in the home environment can significantly limit health self-management and restrict participation at home, and in social and community activities [45–47]. The most common home modifications include installing a wheelchair-accessible shower, grab bars near the toilet, ramps, and a wheelchair-accessible kitchen worktop [44]. In Korea, financial support for home modification following a SCI is available, but it is contingent upon the individuals’ income and subject to a maximum funding limit per person. To address this issue, providing adequate funding for home modifications, adjusting building policies to promote accessibility, increasing the availability of accessible housing options, and providing education to healthcare professionals on the distribution and utilization of home modifications could significantly improve accessibility and quality of life for individuals with SCI.

While the highest priority needs for support services are mostly related to essential economic needs, the second top priorities focus on cultivating the necessary life skills for functioning at home, at work, and within the community. The services include “training for daily living activities” and “vocational rehabilitation”. SCI often requires significant functional alterations [48], profoundly impacting individuals’ engagement in ADL and a job [49]. Understanding these functional changes (e.g., diminished mobility capability) is a high priority due to the intricate arrangements individuals with SCI need to maintain independence [50]. Consequently, individuals with SCI may require instruction in new techniques and how to use assistive devices to compensate for reduced muscle strength, limited range of motion, or diminished endurance [51]. Adequate support is also essential to perform daily activities and maintain productivity at work. As age-related changes intersect with SCI-related functional alterations, individuals may experience shifts in their approaches to ADLs and work ability, leading to an increased reliance on additional assistance and specialized equipment over time to maintain independence [50]. Addressing their changing needs requires ongoing functional support services tailored to the specific physical changes they encounter, especially considering the extended life expectancy of this population. Services should also provide continuous training and support to optimize independence and quality of life across the lifespan.

Although our study sheds light on the priorities for support services for individuals with SCI, it is important to acknowledge the limitations. First, due to data availability constraints, we were unable to delve into the specific needs of SCI support services, such as neurologic bladder and bowel management, sexuality and fertility management, and spasticity management. To address this gap, future research should conduct focus group interviews for comprehensive needs assessment specifically tailored to the SCI population. Another limitation of our study is that the sample was exclusively drawn from members of the KSCIA. As a result, the findings may reflect the specific perspectives and experiences of KSCIA members, potentially introducing selection bias. Individuals who are not members of KSCIA, including those who may not actively seek organizational support or resources, were not included in the study. This limits the generalizability of the results to the broader population of individuals with SCI in South Korea. Future research should aim to include a more diverse and representative sample to enhance the accuracy and applicability of the findings.

## Conclusions

This study identified and prioritized the support service needs of individuals with SCI in South Korea, highlighting critical areas requiring attention. Our analysis, integrating both the Borich needs assessment and the locus for focus model, revealed that the top priorities are “support for caregiver costs,” “livelihood security,” “home modification,” and “health management,” which underscores the multifaceted nature of support needs among individuals with SCI. The findings also emphasize the significance of addressing not only their medical requirements but also broader socio-economic factors that profoundly impact their quality of life. These findings highlight the critical importance of addressing the diverse and complex support service needs of individuals with SCI. By identifying and prioritizing key areas for intervention, healthcare providers and policymakers can better allocate resources and develop targeted strategies to enhance the overall well-being and successful community integration of individuals living with SCI.

## Data Availability

The datasets generated and/or analyzed in the current study are available in the repository of the Korea Spinal Cord Injury Association, where they will be provided upon request: http://www.kscia.org/kscia/.
